# Enhancing Near‐Room‐Temperature Thermoelectric Performance of n‐Type Mg_3_(Sb, Bi)_2_‐Based Materials via ZrB_2_ Heterointerface Engineering

**DOI:** 10.1002/advs.77148

**Published:** 2026-08-13

**Authors:** Yangyang Xu, Li Zhang, Meng Li, Jie Zhang, Nan‐Hai Li, Yan‐Ling Yang, Xiao‐Lei Shi, Zhi‐Gang Chen

**Affiliations:** ^1^ School of Materials Science and Engineering Shaanxi University of Science & Technology Xi'an People's Republic of China; ^2^ School of Chemistry and Physics, ARC Research Hub in Zero‐emission Power Generation for Carbon Neutrality, and Centre for Materials Science Queensland University of Technology Brisbane Queensland Australia

**Keywords:** Mg_3_(Sb, Bi)_2_, interface, material, thermoelectric, ZrB_2_

## Abstract

Mg_3_(Sb, Bi)_2_‐based thermoelectric materials have shown considerable potential for low‐grade waste‐heat recovery near room temperature. However, their thermoelectric performance remains limited by the strong coupling among electrical conductivity, Seebeck coefficient, and thermal conductivity. Here, a conductive ceramic composite strategy based on ZrB_2_ is proposed to construct stable heterointerfaces within an Mg_3.4_Bi_1.29_Sb_0.7_Te_0.01_ matrix, thereby enabling the synergistic regulation of interfacial charge distribution, carrier transport, and phonon scattering. First‐principles calculations reveal that the work‐function difference between Mg_3_(Sb, Bi)_2_ and ZrB_2_ drive interfacial charge redistribution and generate a localized built‐in electric field at the contact region. Experimental results demonstrate that appropriate incorporation of ZrB_2_ significantly increases carrier concentration and electrical conductivity while maintaining relatively high carrier mobility. Compared with the pristine matrix, the 1.0 wt.% ZrB_2_ composite sample exhibits substantially enhanced room‐temperature thermoelectric performance. Specifically, the room‐temperature power factor increases from 19.11 to 28.51 µW cm^−1^ K^−2^ and reaches 30.82 µW cm^−1^ K^−2^ at 373 K. Meanwhile, a maximum *ZT* value of 1.22 is achieved at 573 K, and the corresponding single‐leg theoretical maximum conversion efficiency reaches 12.92% under a temperature difference of 470 K, demonstrating conductive ceramic heterointerface engineering as an effective strategy for enhancing the thermoelectric performance of Mg_3_(Sb, Bi)_2_‐based materials.

## Graphical abstract

A conductive ZrB_2_ ceramic composite strategy was developed for Mg_3_(Sb, Bi)_2_ thermoelectrics to regulate carrier transport and phonon scattering through heterointerface engineering. The 1.0 wt.% ZrB_2_ composite achieved enhanced room‐temperature performance, with a power factor of 28.51 µW cm^−1^ K^−2^ and a peak *ZT* of 1.22 at 573 K.

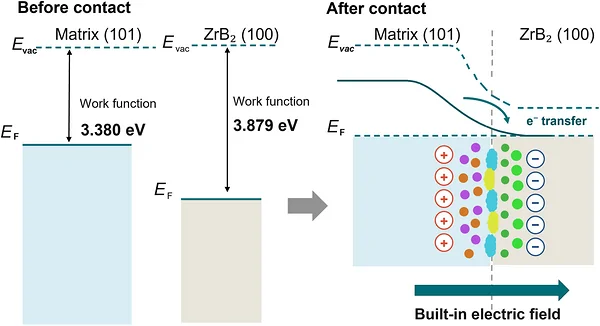

## 1 Introduction

With the continuous growth of global energy demand and the increasing need for low‐grade heat utilization, the development of efficient, reliable, and environmentally friendly energy‐conversion technologies has become an important research direction in the field of energy materials [1, 2, 3, 4, 5]. Thermoelectric technology enables direct and reversible conversion between thermal energy and electrical energy, offering advantages such as the absence of moving mechanical parts, low maintenance cost, vibration‐free operation, and facile miniaturized integration [6, 7]. Consequently, it exhibits significant application potential in waste‐heat recovery, localized cooling, and solid‐state thermal management [8, 9, 10, 11, 12]. The energy‐conversion performance of thermoelectric materials is generally evaluated by the dimensionless thermoelectric figure of merit, *ZT*, defined as *ZT* = *S* ^2^ *σT*/(*κ*_L_ + *κ*_e_) = *S* ^2^ *σT*/*κ*, where *S* is the Seebeck coefficient, *σ* is the electrical conductivity, *S* ^2^ *σ* is the power factor, *T* is the absolute temperature, *κ*_L_ is the lattice thermal conductivity, *κ*_e_ is the electronic thermal conductivity, and *κ* is the total thermal conductivity [3, 13]. The practical performance of thermoelectric devices fundamentally depends on the average *ZT* (*ZT*_ave_) and the engineering *ZT* within the target operating temperature range [14].

Although conventional thermoelectric materials such as Bi_2_Te_3_ [15], PbSe [16], and SnTe [16] have demonstrated excellent performance near room temperature and in the mid‐temperature range, issues including the scarcity of Te resources, the toxicity of Pb, and the volatility of certain chalcogen elements at elevated temperatures significantly hinder their sustainable development and large‐scale applications [17]. In contrast, Mg_3_(Sb, Bi)_2_‐based Zintl‐phase materials, which possess advantages such as low cost, low toxicity, and earth‐abundant constituent elements, have rapidly emerged as one of the most promising next‐generation thermoelectric material systems [18, 19]. Since their initial discovery, Mg_3_(Sb, Bi)_2_ Zintl compounds have been regarded as promising alternatives to conventional Bi_2_Te_3_‐based thermoelectric materials because of their non‐toxicity, environmental friendliness, and abundant elemental reserves [20, 21]. Meanwhile, the complex chemical bonding characteristics and significant atomic mass differences within this system are favorable for enhancing phonon scattering, thereby endowing the materials with intrinsically low *κ*_L_ [21, 22, 23].

Intrinsic Mg_3_Sb_2_ readily forms Mg vacancies and typically exhibits low‐mobility p‐type transport behavior, which severely limits its thermoelectric performance [24, 25]. Through strategies such as excess Mg regulation and donor doping with Te or rare‐earth/transition‐metal elements, researchers have successfully realized n‐type Mg_3_Sb_2_‐based materials and significantly improved their electrical transport properties [26, 27, 28]. Furthermore, the introduction of Mg_3_Bi_2_ to construct n‐type Mg_3_(Sb, Bi)_2_ solid solutions not only enables band‐structure modulation, bandgap narrowing, and effective‐mass optimization, but also enhances the application potential of this system from near‐room temperature to the mid‐temperature range [29]. Extensive efforts have been devoted to optimizing n‐type Mg_3_(Sb, Bi)_2_‐based thermoelectric materials, including studies on Mg volatilization and migration [30, 31, 32], grain‐size optimization [33], sintering‐thickness regulation [34], defect engineering [35, 36, 37, 38], micro/nanostructure modulation [39], as well as the non‐uniform characteristics and resistance optimization of grain boundaries [24, 32, 40, 41]. Although these studies have substantially advanced the development of Mg_3_(Sb, Bi)_2_‐based materials, further enhancement of their thermoelectric performance near room temperature remains constrained by multiple factors [42]. In the near‐room‐temperature region, carrier mobility (*µ*) is highly susceptible to grain‐boundary scattering, defect distribution, and microstructural inhomogeneity, thereby limiting further improvement of the *S* ^2^ *σ* [43]. Therefore, how to effectively regulate carrier concentration (*n*) without significantly sacrificing *µ*, while simultaneously enhancing phonon scattering, remains a central challenge for achieving high‐performance Mg_3_(Sb, Bi)_2_‐based thermoelectric materials [44].

Among the various performance‐optimization strategies, introducing functional secondary phases to construct composite systems has been widely regarded as an effective approach for achieving synergistic regulation of electron and phonon transport [45, 46]. Through the rational selection of secondary phases, it is possible not only to modulate carrier transport behavior and optimize interfacial energy‐filtering effects, but also to enhance the scattering of mid‐ to high‐frequency phonons via heterointerfaces and the associated defects, such as nanoprecipitates and dislocations, thereby further reducing the *κ*_L_ [45, 47]. Compared with conventional metallic secondary phases, refractory ceramic secondary phases are more suitable for Mg_3_(Sb, Bi)_2_‐based composite systems because of their superior thermal stability and tunable heterointerface/grain‐boundary structures, which enable simultaneous regulation of structural stability and coupled electron‐phonon transport [32, 43, 48]. In this context, ZrB_2_, a transition‐metal boride possessing high electrical transport capability, a high melting point, and excellent chemical stability, provides a promising candidate for composite modification of Mg_3_(Sb, Bi)_2_ matrices [49, 50, 51]. The incorporation of ZrB_2_ is expected to induce localized charge redistribution through the formation of heterointerfaces, thereby optimizing electrical transport properties, while simultaneously enhancing interfacial phonon scattering. Such effects are anticipated to enable synergistic enhancement of the *S* ^2^ *σ* and suppression of *κ*_L_. Therefore, this work focuses on the Mg_3_(Sb, Bi)_2_/ZrB_2_ composite system and systematically investigates the heterointerface effects induced by the ZrB_2_ secondary phase, as well as their influence on carrier transport and phonon‐scattering mechanisms. By establishing the correlations among interfacial structure, electronic regulation, and thermoelectric performance, this study provides new insights into interface engineering and performance optimization for high‐performance n‐type Mg_3_(Sb, Bi)_2_‐based thermoelectric materials operating near room temperature.

## 2 Results and Discussion

In this work, Mg_3.4_Bi_1.29_Sb_0.7_Te_0.01_ was selected as the matrix phase because, based on both our previous studies and reports from other researchers, materials with this composition generally exhibit relatively stable and favorable initial thermoelectric performance [42, 52]. Based on this, to clarify the influence of the ZrB_2_ secondary phase on the transport behavior of the Mg_3.4_Bi_1.29_Sb_0.7_Te_0.01_ matrix, first‐principles calculations were first performed to construct an Mg_3_(Sb, Bi)_2_(101)/ZrB_2_(100) heterointerface model. The corresponding work function, structural relaxation, differential charge density, electron localization function (ELF), and density of states (DOS) characteristics were systematically analyzed. The work function was calculated from the energy difference between the vacuum level and the Fermi level in the surface model, according to *Φ* = *E*_vac_ − *E*_f_, where *E*_vac_ represents the vacuum energy level corresponding to the plateau region of the planar‐averaged electrostatic potential along the surface‐normal direction, and *E*_f_ is the Fermi level of the system [53]. As shown in Figure 1a, the calculated work function of the Mg_3_(Sb, Bi)_2_ (101) surface is 3.380 eV, whereas that of the ZrB_2_ (100) surface is 3.879 eV. After contact between the two phases, electrons tend to transfer from the Mg_3_(Sb, Bi)_2_ side with the lower work function to the ZrB_2_ side with the higher work function until the *E*_f_ on both sides of the interface reach equilibrium. Considering the complexity of the actual alloy solid‐solution microstructure, pure Mg_3_Bi_2_ was employed as a representative matrix model in the theoretical simulations to optimize computational efficiency while preserving the essential electronic‐structure characteristics. Accordingly, an Mg_3_Bi_2_(101)/ZrB_2_(100) heterointerface model was established for subsequent calculations.

**FIGURE 1:**
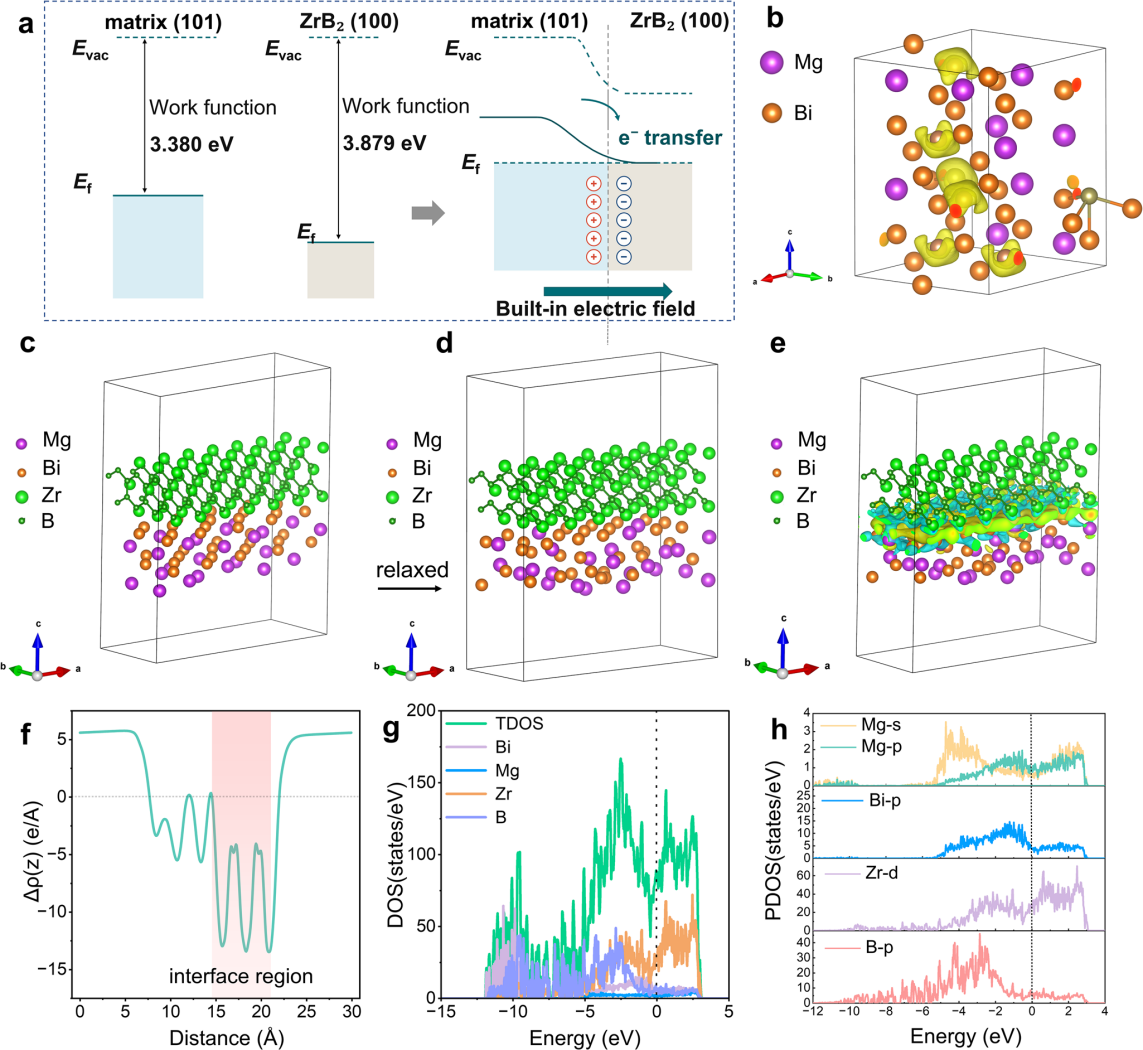
First‐principles investigation of interfacial charge redistribution and electronic‐structure modulation at the Mg_3_Bi_2_/ZrB_2_ heterointerface. (a) Work‐function alignment and schematic illustration of electron transfer between the Mg_3_Bi_2_(101) and ZrB_2_(100) surfaces. Here *E*_vac_ represents the vacuum energy level corresponding to the plateau region of the planar‐averaged electrostatic potential along the surface‐normal direction, and *E*_f_ is the Fermi level of the system. (b) Electron localization function (ELF) of the Mg_3_Bi_2_ matrix. (c,d) Atomic configurations of the heterointerface before and after structural relaxation, respectively. (e) Three‐dimensional (3D) differential charge‐density distribution of the relaxed interface, where the yellow and cyan regions represent charge accumulation and charge depletion, respectively (isosurface value: 0.0017 e Å^−3^). (f) Planar‐averaged differential charge density along the interfacial normal direction. (g) Total and element‐projected density of states (DOS) of the heterointerface system. (h) Orbital‐resolved projected density of states (PDOS) of representative Mg, Bi/Sb, Zr, and B atoms.

The ELF shown in Figure 1b further reflects the localized electronic bonding and charge distribution within the Mg_3_Bi_2_ matrix. Electrons are mainly localized around the Bi/Sb atoms, whereas the degree of electron localization near the Mg atoms is relatively weak, indicating the presence of pronounced charge‐transfer characteristics within the Mg_3_Bi_2_ matrix. This electronic distribution provides the electronic‐structure basis for interfacial charge redistribution upon contact with ZrB_2_. Figure 1c,d presents the construction and structural optimization results of the ZrB_2_(100)/Mg_3_Bi_2_(101) heterointerface model, respectively. It can be observed that the ZrB_2_ secondary phase forms a relatively continuous interfacial contact with the Mg_3_Bi_2_ matrix, without obvious structural collapse or severe lattice distortion at the interface, indicating that the heterointerface is structurally feasible [45]. After structural optimization, local atomic relaxation occurs near the interface, suggesting the existence of stable interfacial interactions between the two phases rather than simple mechanical mixing [54]. As shown in Figure 1e, distinct charge‐accumulation and charge‐depletion regions emerge at the interface. To better illustrate the structural details of this region, an enlarged view is provided in Figure S1. Correspondingly, the planar‐averaged differential charge‐density curve in Figure 1f exhibits strong fluctuations within the contact region, demonstrating that charge transfer is mainly concentrated near the ZrB_2_/Mg_3_Bi_2_ heterointerface [55]. Such localized charge redistribution can generate a built‐in electric field near the interface, thereby influencing carrier distribution and transport behavior.

The DOS results further support the interfacial electronic‐coupling mechanism. As shown in Figure 1g, the heterointerface system exhibits pronounced electronic‐state contributions near the *E*_f_, indicating that the interface retains favorable electronic transport capability [56]. The element‐projected DOS reveals that the electronic states near the *E*_f_ mainly originate from Zr/B‐related states and Bi/Sb‐related states, whereas the contribution from Mg is relatively weak. The orbital‐projected DOS shown in Figure 1h demonstrates that the Zr‐*d* and B‐*p* orbitals overlap within a similar energy range, reflecting the strong Zr─B orbital hybridization within ZrB_2_, which is an important origin of its metallic‐like conductive behavior. Meanwhile, the Bi/Sb‐*p* states exhibit energy overlap with the Zr/B‐related states near the *E*_f_, suggesting the existence of electronic‐structure coupling at the interface region. Collectively, these results indicate that the ZrB_2_ heterointerface can regulate carrier distribution through both work‐function differences and interfacial electronic‐state coupling, thereby providing a mechanistic basis for the experimentally observed enhancement in *n* and *S* ^2^ *σ*.

To experimentally verify the interfacial charge transfer and local coordination modulation predicted by the above first‐principles calculations, synchrotron X‐ray absorption fine structure (XAFS) spectroscopy combined with two‐dimensional (2D) wavelet transform (WT) analysis was further employed to investigate the electronic structure and local environment of Bi atoms in the Mg_3.4_Bi_1.29_Sb_0.7_Te_0.01_‐1.0 wt.% ZrB_2_ composite. As shown in Figure 2a, the normalized Bi L_3_‐edge XANES spectra reveal a slight shift in the absorption‐edge position for the composite sample compared with the Mg_3.4_Bi_1.29_Sb_0.7_Te_0.01_ matrix, indicating that the local electronic states surrounding the Bi atoms are weakly modulated after the introduction of ZrB_2_. This variation suggests the possible existence of interfacial charge redistribution induced by the work‐function difference, which is consistent with the previously calculated differential charge‐density results. Furthermore, the 2D WT contour maps (Figure 2b,c) together with the Fourier‐transformed k^2^‐weighted EXAFS radial structural spectra (Figure 2d) further reveal subtle changes in the local coordination environment around Bi atoms. The major scattering‐intensity centers of the composite sample (Figure 2b) and the pristine matrix sample (Figure 2c) remain generally consistent in both k‐space and R‐space, indicating that the introduction of the ZrB_2_ secondary phase does not significantly disrupt the primary local structure of the Mg_3_(Bi, Sb)_2_ matrix. At the same time, the WT features of both the composite and matrix samples are clearly distinct from those of the Bi foil (Figure 2e) and Bi_2_O_3_ reference samples (Figure 2f), and no characteristic scattering signals corresponding to metallic Bi or Bi─O coordination environments are observed. This result confirms that no obvious precipitation of elemental Bi or formation of Bi oxide by‐products occurs during the composite fabrication process. Notably, Figure 2b,c exhibits discernible differences in the local scattering intensity and contour distribution within the high‐k/high‐R region in the upper‐right corner, suggesting that the higher‐order coordination or multiple‐scattering characteristics surrounding Bi atoms are slightly modulated after the incorporation of ZrB_2_. Such local variations can be attributed to the local coordination perturbation around Bi induced by lattice distortion and charge redistribution at the heterointerface, rather than substantial changes in the bulk crystal structure. Collectively, these experimental results support the theoretically predicted stable and feasible relaxed Mg_3_(Bi, Sb)_2_/ZrB_2_ heterointerface structure and further indicate that the interfacial built‐in electric field together with local lattice distortion may jointly participate in regulating carrier scattering and transport behavior.

**FIGURE 2:**
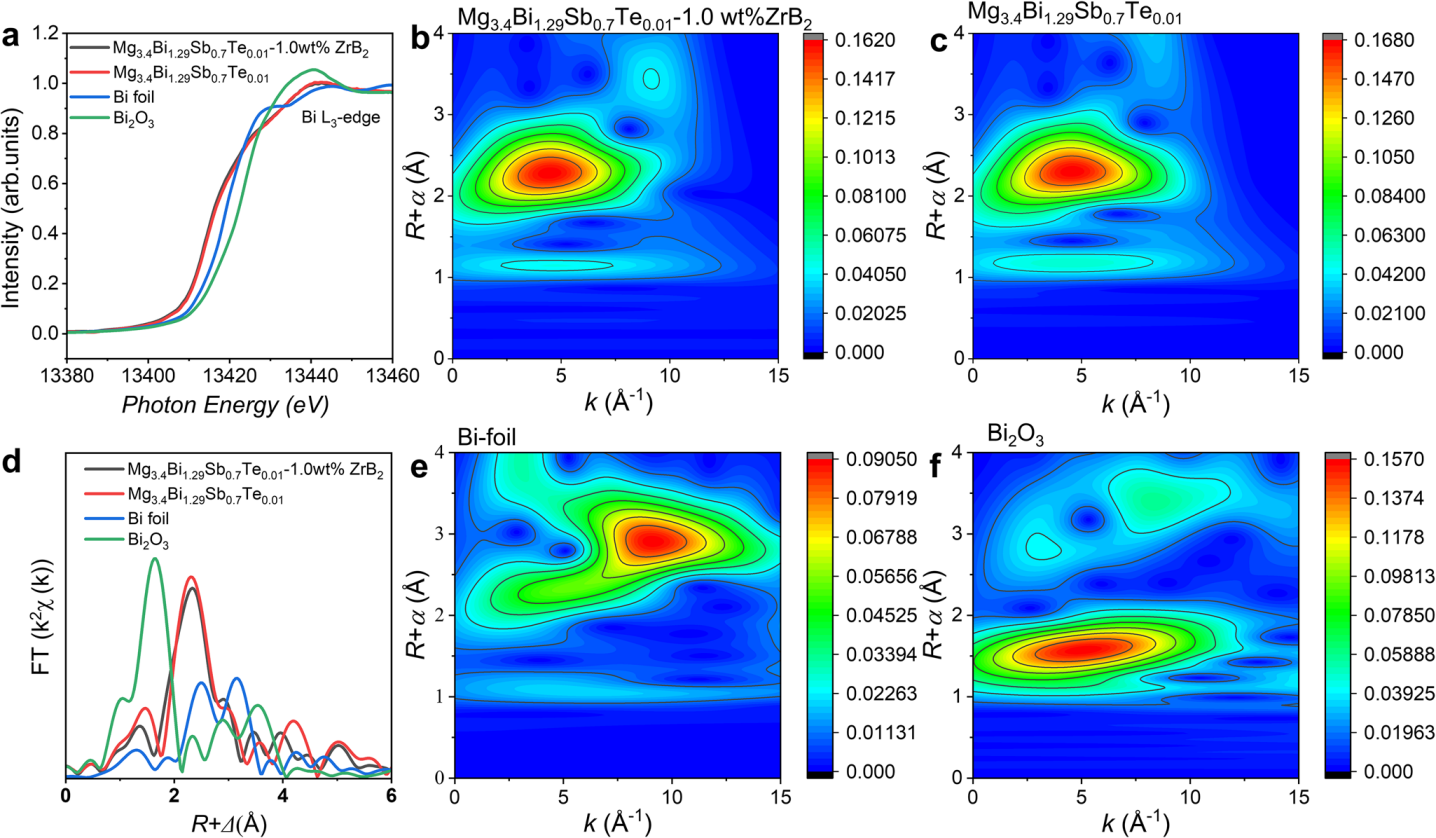
Bi L_3_‐edge synchrotron X‐ray absorption fine structure (XAFS) analysis of Mg_3.4_Bi_1.29_Sb_0.7_Te_0.01_ and Mg_3.4_Bi_1.29_Sb_0.7_Te_0.01_‐1.0wt.%ZrB_2_. (a) Normalized Bi L_3_‐edge XANES spectra of Mg_3.4_Bi_1.29_Sb_0.7_Te_0.01_, Mg_3.4_Bi_1.29_Sb_0.7_Te_0.01_‐1.0wt.%ZrB_2_, ZrB_2_, Bi foil, and Bi_3_O_3_. (b,c) Wavelet transform (WT)‐EXAFS contour plots of Mg_3.4_Bi_1.29_Sb_0.7_Te_0.01_‐1.0wt.%ZrB_2_ and Mg_3.4_Bi_1.29_Sb_0.7_Te_0.01_, respectively. (d) Fourier‐transformed k^2^‐weighted EXAFS spectra in R‐space without phase‐shift correction. (e,f) WT‐EXAFS contour plots of Bi foil and Bi_2_O_3_ references, respectively. Here, *k* denotes the photoelectron wave vector; *R* denotes the radial distance from the absorbing Bi atom; FT denotes Fourier transform; *R* + *Δ* represents the apparent radial distance in the Fourier‐transformed EXAFS spectra, where Δ is the phase‐shift‐induced distance correction; and *R* + *α* denotes the apparent radial distance in the WT‐EXAFS plots, where α represents the phase‐shift‐related offset.

To investigate the influence of ZrB_2_ content on the thermoelectric performance and to determine the optimal ZrB_2_ concentration, a series of Mg_3.4_Bi_1.29_Sb_0.7_Te_0.01_‐*x* wt.% ZrB_2_ (*x* = 0, 0.5, 1.0, and 1.5) samples were designed and fabricated. A series of structural characterizations was subsequently carried out on the prepared samples. Figure 3a presents the XRD patterns collected from powder samples. The major diffraction peaks of all samples can be well indexed to the standard PDF card of Mg_3_Bi_2_ (Mg_3_Bi_2_#04‐0464), indicating that the composite samples remain predominantly composed of the Mg_3_(Bi, Sb)_2_ solid‐solution phase after ZrB_2_ incorporation, and that the introduction of a small amount of ZrB_2_ does not significantly disrupt the crystal structure of the matrix. Owing to the relatively low content of ZrB_2_ and its dispersion within the matrix, the characteristic diffraction peaks of ZrB_2_ are difficult to clearly distinguish in conventional XRD patterns. The enlarged view in Figure 3b shows that the diffraction peaks of the matrix exhibit slight shifts with increasing ZrB_2_ content, suggesting that the introduction of the secondary phase influences the local lattice environment of the matrix through interfacial strain or local compositional fluctuations. Furthermore, as shown in Figure 3c, the lattice parameter a exhibits an overall slight decrease, whereas the *c*‐axis parameter first increases and then decreases with increasing ZrB_2_ content. These results indicate that the incorporation of ZrB_2_ induces a subtle anisotropic effect on the lattice of the Mg_3_(Bi, Sb)_2_ matrix. This observation further suggests that ZrB_2_ mainly exists as a secondary phase while simultaneously introducing slight anisotropic lattice modulation to the matrix through heterointerface interactions.

**FIGURE 3:**
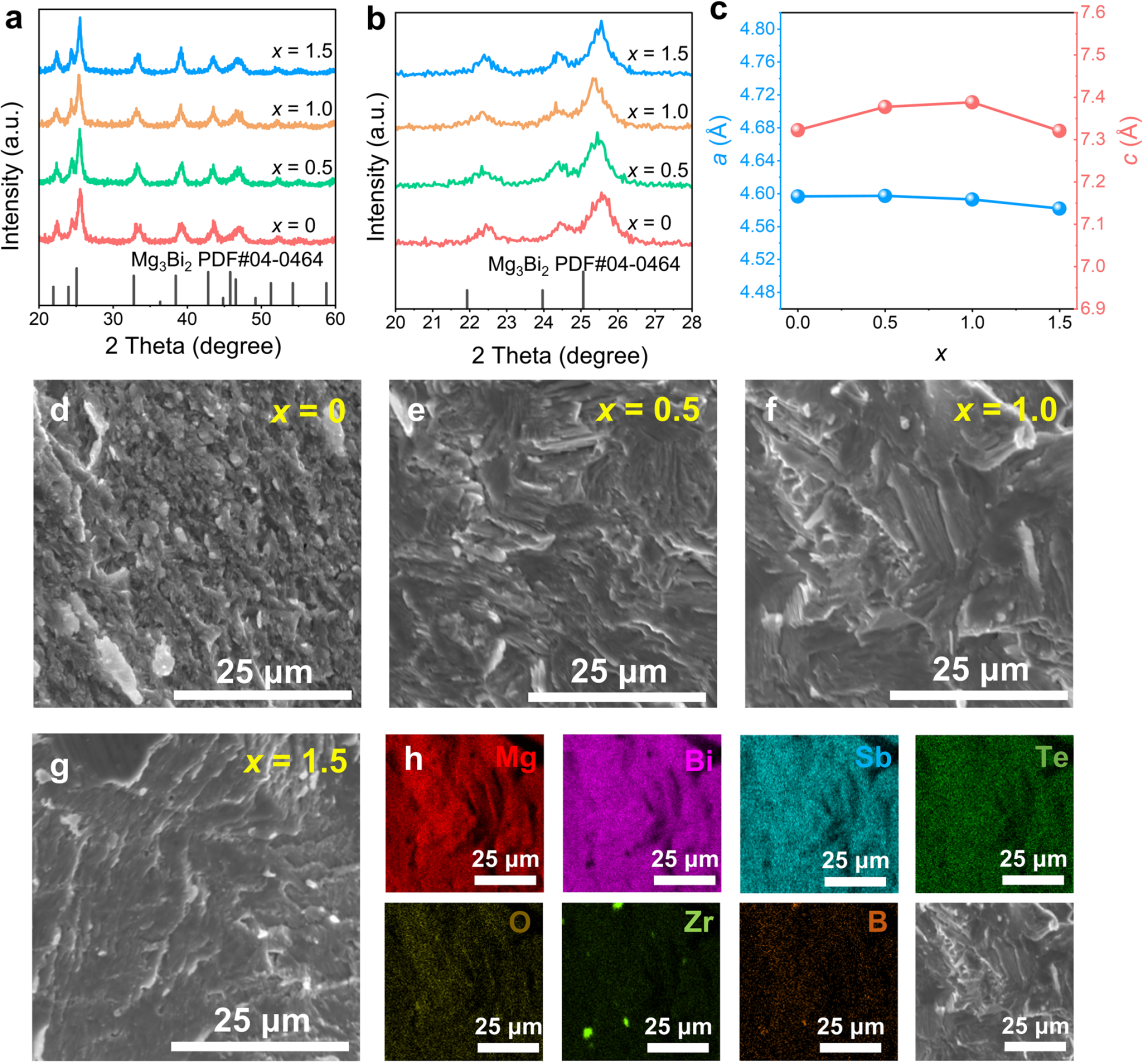
Structural characterizations of Mg_3.4_Bi_1.29_Sb_0.7_Te_0.01_‐*x* wt.% ZrB_2_ (*x* = 0, 0.5, 1.0, and 1.5). (a) X‐ray diffraction (XRD) patterns collected in the 2*θ* range of 20° to 60°; (b) Magnified XRD patterns in the 2*θ* range of 20° to 28°; (c) Evolution of lattice parameters *a* and *c* with ZrB_2_ content. PDF: powder diffraction file. (d–g) Scanning electron microscopy (SEM) images of fresh fracture surfaces. (h) Energy dispersive X‐ray spectroscopy (EDS) elemental mappings of Mg, Bi, Sb, Te, O, Zr and B for the *x* = 1.0 sample. The corresponding SEM image of the mapped region is shown in the lower‐right corner.

Figure 3d–g present the freshly fractured surface morphologies of the Mg_3.4_Bi_1.29_Sb_0.7_Te_0.01_‐*x* wt.% ZrB_2_ (*x* = 0, 0.5, 1.0, and 1.5) samples. No obvious cracks or large pores are observed in any sample, which is consistent with the density measurement results shown in Table S1 and indicates that the composite samples possess very high densification. Although ZrB_2_ has a theoretical density of 6.09 g cm^−3^, the relative density of the composite samples varies nonmonotonically with increasing ZrB_2_ content due to the combined effects of particle packing, secondary‐phase distribution, interfacial area, and residual porosity during the sintering process [57]. The pristine sample (*x* = 0) exhibits a relatively rough fracture surface with irregular granular fracture features. After the introduction of ZrB_2_, the fracture morphology gradually evolves into a more pronounced layered and wrinkled structure, particularly in the *x* = 0.5 and *x* = 1.0 samples. This morphological evolution suggests that an appropriate amount of ZrB_2_ may alter the grain‐growth behavior and fracture pathways while introducing more secondary‐phase/matrix heterointerfaces within the matrix. It should be noted that the morphological variation itself cannot directly prove the enhancement of *µ*. However, the heterointerfaces and multiscale microstructures reflected by these features are expected to simultaneously influence electron and phonon transport. On the one hand, appropriately dispersed ZrB_2_ interfaces may regulate the carrier distribution near the interface through localized charge redistribution. On the other hand, the secondary‐phase/matrix interfaces can act as additional phonon‐scattering centers, thereby contributing to the reduction of *κ*_L_. Such structural characteristics provide a microstructural basis for the synergistic optimization between enhanced electrical transport and suppressed thermal transport. To further confirm the spatial distribution of ZrB_2_, EDS elemental mapping was performed on the *x* = 1.0 sample, as shown in Figure 3h. The Mg, Bi, Sb, and Te signals are distributed relatively uniformly, indicating good compositional homogeneity of the matrix. In contrast, the Zr signal appears as localized bright regions, while the spatial distribution of the B signal corresponds well with that of Zr, confirming that the ZrB_2_ secondary phase has been successfully introduced and dispersed within the Mg_3_(Sb, Bi)_2_ matrix. It should be noted that, due to the low atomic number of B and the low energy of its characteristic X‐rays, the EDS signal of B is typically weak. Therefore, the B elemental mapping mainly serves as auxiliary evidence for identifying the distribution tendency of ZrB_2_ particles.

To investigate the interfacial characteristics introduced by ZrB_2_, multiscale transmission electron microscopy (TEM) characterization was further conducted on the *x* = 1.0 sample, focusing on the phase interfaces, grain boundaries, and associated lattice distortions. As shown in Figure 4a, a localized dark‐contrast region is observed in the upper part of focused ion beam (FIB)‐prepared Mg_3_(Sb, Bi)_2_ matrix. The corresponding TEM‐EDS maps in Figure 4b reveal the spatial co‐localization of Zr and B within this region, whereas Mg and Bi are predominantly distributed in the surrounding matrix, identifying the dark‐contrast region as a ZrB_2_ secondary phase. As further shown by the complete elemental maps in Figure S2, Sb and Te are also mainly distributed in the Mg_3_(Sb, Bi)_2_ matrix. These results demonstrate that the introduced ZrB_2_ is retained as a distinct secondary phase. Moreover, no extensive diffusion of Zr or B into the matrix is detectable within the spatial resolution of the EDS analysis, indicating that the ZrB_2_ phase largely preserves its compositional integrity. An MgO phase is also observed in the Mg_3_(Sb, Bi)_2_ matrix, as confirmed by the co‐enrichment of Mg and O in Figure S3. This minor oxide phase may provide additional phonon‐scattering sites [58]. The high‐resolution TEM (HRTEM) image obtained from a matrix grain adjacent to the ZrB_2_ phase is presented in Figure 4c. Continuous lattice fringes and well‐defined diffraction spots in the corresponding selected area electron diffraction (SAED) pattern indicate the high crystallinity of the Mg_3_(Sb, Bi)_2_ matrix. The geometric‐phase analysis (GPA)‐derived strain maps in Figure 4d further reveal a heterogeneous local strain distribution, with alternating tensile and compressive regions. Such local strain fluctuations may originate from intrinsic point defects, substitutional disorder, local compositional variations, and lattice mismatch associated with the secondary phase, thereby introducing additional perturbations to phonon propagation. As shown in Figure 4e, a nonplanar ZrB_2_/matrix interface is observed, together with a thin structurally disordered interfacial transition layer. This irregular interfacial morphology increases the structural discontinuity experienced by propagating phonons and is therefore expected to enhance interfacial phonon scattering. The HRTEM image and indexed SAED pattern of the secondary phase in Figure 4f exhibit well‐resolved lattice fringes and diffraction spots. The measured interplanar spacing of approximately 2.74 Å agrees well with the (100) plane of hexagonal ZrB_2_, further confirming the presence of the introduced ZrB_2_ as a distinct secondary phase in the Mg_3_(Sb, Bi)_2_ matrix. In addition, pronounced local lattice distortions are observed within the Mg_3_(Sb, Bi)_2_ matrix, as shown in Figure 4 g, indicating local deviations from the ideal periodic lattice. The low‐magnification TEM image in Figure 4 h reveals an irregular grain boundary with a rough morphology. Further HRTEM observation in Figure 4i shows the lattice arrangement on both sides of a grain boundary. The fast Fourier transform (FFT) patterns obtained from the boxed regions in Grain 1 and Grain 2 exhibit different lattice orientations, suggesting crystallographic misorientation and lattice discontinuity across the boundary. Collectively, the local strain fields, lattice distortions, ZrB_2_/matrix interface, and rough grain‐boundary morphology constitute a multiscale defect and interfacial architecture. These structural features are expected to provide effective scattering centers for phonons over a broad wavelength range, thereby impeding lattice heat transport.

**FIGURE 4:**
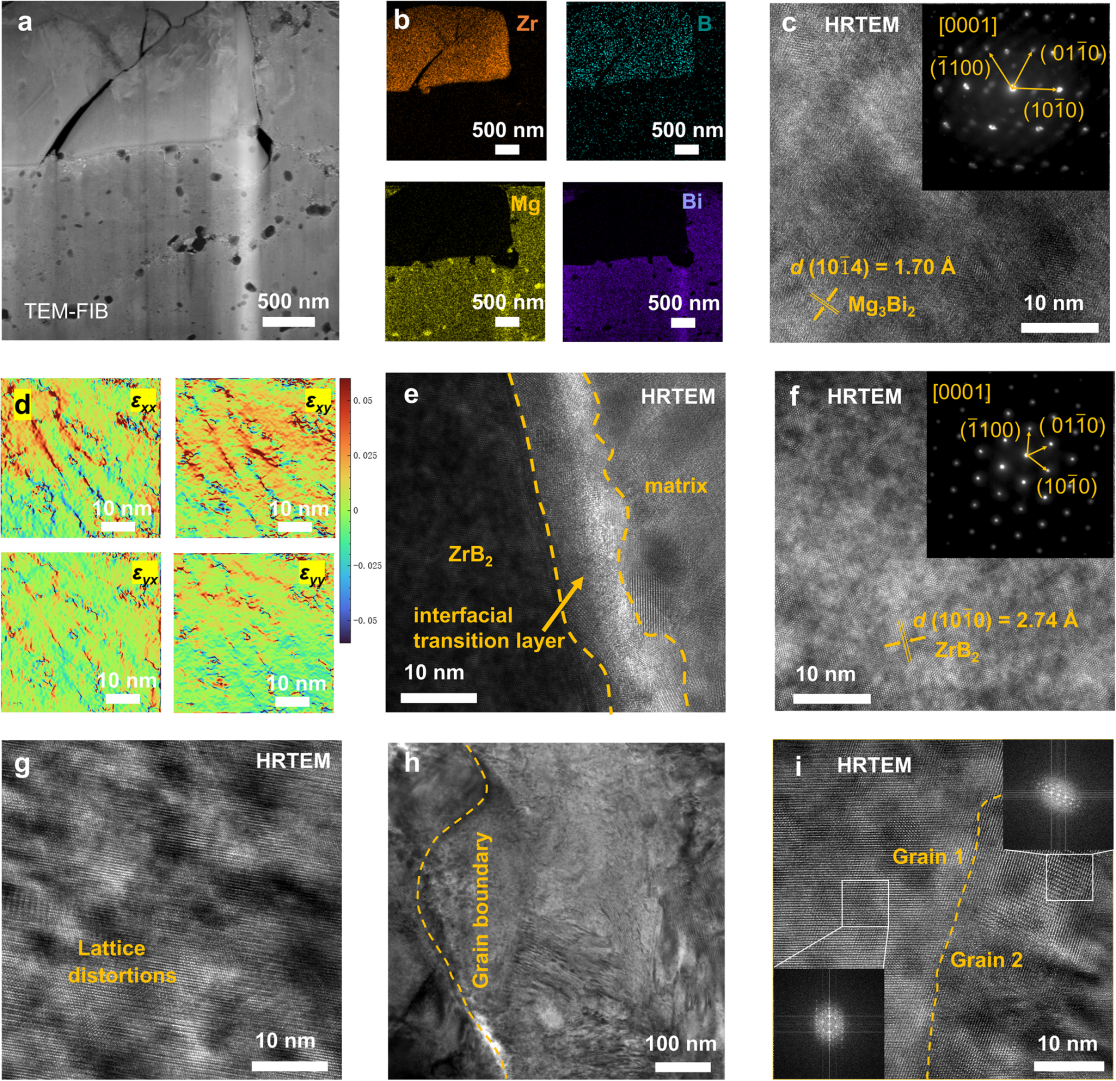
Multiscale transmission electron microscopy (TEM) characterization of the Mg_3.4_Bi_1.29_Sb_0.7_Te_0.01_‐1.0 wt.% ZrB_2_ sample. (a) Low‐magnification TEM image of the focused ion beam (FIB)‐prepared lamella, showing the overall microstructure, grain morphology, and grain‐boundary distribution. (b) Corresponding TEM‐EDS elemental maps of Zr, B, Mg, and Bi obtained from the region shown in (a). (c) High‐resolution TEM (HRTEM) image of the Mg_3_(Sb, Bi)_2_ matrix adjacent to a ZrB_2_ secondary‐phase region. The inset shows the corresponding indexed selected area electron diffraction (SAED) pattern. (d) Local strain maps derived from the HRTEM image in (c) by geometric phase analysis. (e) HRTEM image of the ZrB_2_/matrix interface. (f) HRTEM image of the ZrB_2_ secondary phase. The inset shows the corresponding indexed SAED pattern. (g) Representative local lattice distortions within the Mg_3_(Sb, Bi)_2_ matrix. (h) TEM image showing a representative grain boundary in the Mg_3_(Sb, Bi)_2_​ matrix. (i) HRTEM image showing another representative grain boundary, with the dashed line marking the approximate boundary between Grain 1 and Grain 2. The insets show the fast Fourier transform (FFT) patterns obtained from the corresponding boxed regions in the two adjacent grains.

To investigate the influence of ZrB_2_ incorporation on the thermoelectric performance, the electrical transport properties of Mg_3.4_Bi_1.29_Sb_0.7_Te_0.01_‐*x* wt.% ZrB_2_ (*x* = 0, 0.5, 1.0, and 1.5) composite samples are presented in Figure 5a–f. As shown in Figure 5a, the *σ* of all samples gradually decreases with increasing temperature, exhibiting the typical transport behavior of degenerate semiconductors. Compared with the pristine sample (*x* = 0), which exhibits a *σ* of 678.53 S cm^−1^, the ZrB_2_‐containing composite samples display significantly enhanced *σ* throughout the entire measured temperature range. In particular, the *x* = 1.0 sample reaches a room‐temperature *σ* of 1320.53 S cm^−1^, corresponding to an enhancement of approximately 94.62% relative to the matrix sample. Since *σ* = *neµ*, the increase in *σ* generally originates from variations in *n*, *µ*, or the combined contribution of both. Combined with the Hall measurement results shown in Figure 5d,e, it can be observed that the *n* increases significantly after ZrB_2_ incorporation. At 303 K, the *n* increases from 2.48 × 10^19^ cm^−3^ for the *x* = 0 sample to 4.62 × 10^19^ cm^−3^ for the *x* = 1.0 sample. In contrast, the *µ* does not exhibit a comparable level of enhancement. These results indicate that the improvement in *σ* mainly originates from the substantial increase in *n*. This observation is consistent with the interfacial charge redistribution revealed in Figure 1e, where the work‐function difference at the Mg_3_(Sb, Bi)_2_/ZrB_2_ heterointerface induces electron redistribution and localized built‐in electric fields, thereby regulating the carrier distribution near the interface and increasing the effective electron concentration participating in electrical transport.

**FIGURE 5:**
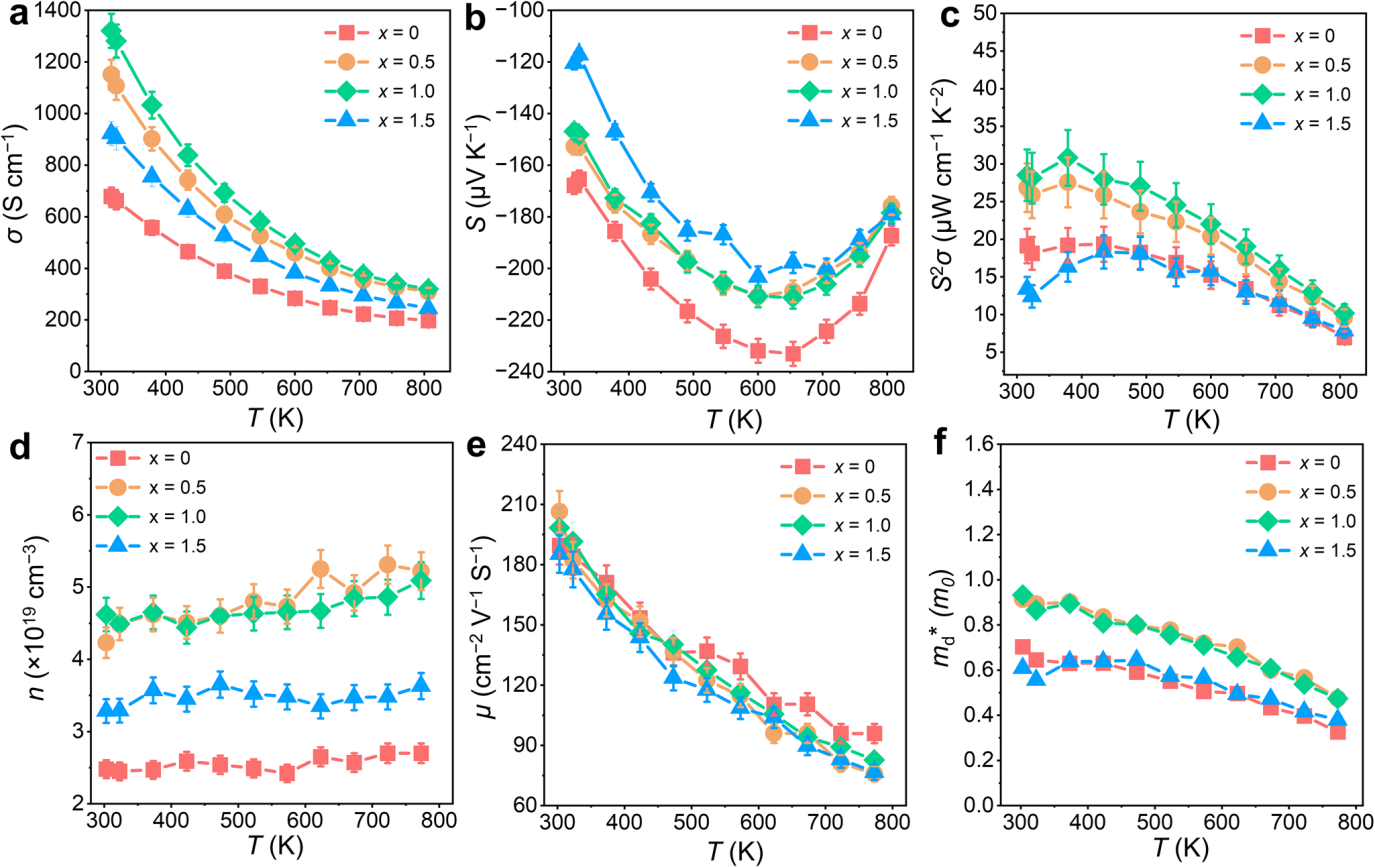
Temperature‐dependent electric transport properties of Mg_3.4_Bi_1.29_Sb_0.7_Te_0.01_‐*x* wt.%ZrB_2_ (*x* = 0, 0.5, 1.0 and 1.5). (a) electrical conductivity (*σ*). (b) Seebeck coefficient (*S*). (c) Power factor (*S* ^2^ *σ*). (d) Carrier concentration (*n*) and (e) mobility (*µ*). (f) Normalized density‐of‐states effective mass (*m*_d_*).

Figure 5b shows that all samples exhibit negative *S*, indicating that the composite materials retain n‐type conduction after ZrB_2_ incorporation. In general, an increase in *n* tends to reduce the absolute value of the *S*. However, the *x* = 0.5 and *x* = 1.0 samples still maintain relatively high |*S*| values, suggesting that the enhancement in *σ* within the composite system is not solely derived from increased *n*. Combined with the results shown in Figure 5f, the normalized density‐of‐states effective mass (*m*_d_*) of the ZrB_2_ composite samples is generally higher than that of the pristine sample, indicating that the introduction of heterointerfaces may effectively modulate the electronic DOS. A higher *m*_d_* is beneficial for maintaining a relatively large *S* even at elevated *n*, thereby alleviating the intrinsic trade‐off between *n* and *S*. As a result, the material can achieve high *σ* while preserving its thermoelectric potential to the greatest extent possible [22].

There exists a competitive relationship between interfacial regulation and interfacial scattering. An appropriate amount of ZrB_2_ can compensate for part of the detrimental influence of interface scattering through the *n* enhancement induced by interfacial charge redistribution, thereby improving the overall electrical transport performance. However, the secondary‐phase/matrix interfaces also increase the probability of carrier scattering, which imposes a certain limitation on *µ* [59]. When the ZrB_2_ content is further increased to 1.5 wt.%, the excessive secondary phase may lead to local agglomeration, an overly high intensity of interfacial scattering centers, or disruption of carrier transport pathways, resulting in the simultaneous deterioration of *n* and *µ* [60]. Benefiting from the synergistic effects of enhanced *n* and optimized electronic DOS, the *x* = 1.0 sample achieves the optimal *S* ^2^ *σ*, as shown in Figure 5c. Compared with the pristine sample (*x* = 0), which exhibits a room‐temperature *S* ^2^ *σ* of 19.11 µW cm^−1^ K^−2^, the incorporation of an appropriate amount of ZrB_2_ significantly enhances the *S* ^2^ *σ* of the composite samples. In particular, the *x* = 1.0 sample reaches a room‐temperature *S* ^2^ *σ* of 28.51 µW cm^−1^ K^−2^, representing an enhancement of approximately 49.19% relative to the matrix sample. Meanwhile, this sample achieves the highest *S* ^2^ *σ* value of 30.82 µW cm^−1^ K^−2^ at 373 K.

To quantitatively evaluate the relationship between the measured *n* and the electrical transport properties, the *n*‐dependent *S* ^2^ *σ* was calculated using the single parabolic band (SPB) model [13, 61, 62], as shown in Figure S4. At 303 K, the measured *n* of the *x* = 1.0 sample is 4.62×10^19^ cm^−3^, which lies on the high‐*n* side of the calculated optimum of approximately 3.0 × 10^19^ cm^−3^. Therefore, a moderate decrease in *n* would shift the sample closer to the theoretical *S* ^2^ *σ* maximum. Nevertheless, the experimental *S* ^2^ *σ* of 28.51 µW cm^−1^ K^−2^ is only approximately 1.12% lower than the calculated maximum value of 28.83 µW cm^−1^ K^−2^, indicating that the *n* already falls within the broad high‐*S* ^2^ *σ* region at room temperature. At 523 K, the measured *n* remains nearly unchanged at 4.63 × 10^19^ cm^−3^, whereas the calculated optimal *n* increases to approximately 8.0 × 10^19^ cm^−3^. The measured value therefore lies on the low‐*n* side of the theoretical optimum, suggesting that a further increase in *n* would be required to approach the maximum *S* ^2^ *σ* at this temperature. However, the experimental *S* ^2^ *σ* of 24.52 µW cm^−1^ K^−2^ is only approximately 3.55% lower than the calculated maximum value of 25.43 µW cm^−1^ K^−2^, confirming that the *x* = 1.0 sample remains within a favorable high‐*S* ^2^ *σ* range. These results demonstrate that the *n* of the *x* = 1.0 sample is close to, although not exactly at, the theoretical *S* ^2^ *σ* optimum over the investigated temperature range. Notably, the direction required for further *n* optimization changes from a moderate decrease at 303 K to an increase at 523 K because the theoretically optimal *n* shifts toward higher values with increasing temperature.

The incorporation of an appropriate amount of ZrB_2_ not only serves as a microstructural modulation phase, but also constructs heterointerfaces featuring charge redistribution characteristics, thereby effectively balancing the intrinsic trade‐off between *σ* and the *S* and ultimately achieving comprehensive optimization of the electrical transport performance of Mg_3_(Sb, Bi)_2_‐based thermoelectric materials. These results demonstrate that the ZrB_2_ secondary phase not only modifies the microstructure of the matrix, but also effectively enhances *n* and optimizes electrical transport behavior through the formation of heterointerfaces with interfacial charge redistribution characteristics. Overall, the charge transfer, built‐in electric field formation, and interfacial electronic‐structure modulation induced by the ZrB_2_/Mg_3_(Sb, Bi)_2_ heterointerfaces are considered to be the major origins of the enhanced *n* and *S* ^2^ *σ* in the composite samples.

Regarding the thermal transport properties, Figure 6a shows that the *κ* of the Mg_3.4_Bi_1.29_Sb_0.7_Te_0.01_‐*x* wt.%ZrB_2_ (*x* = 0, 0.5, 1.0, and 1.5) samples first decreases and then increases with increasing temperature. The *κ* was determined according to *κ* = *DρC*_p_ [63], where *D* is the measured thermal diffusivity in Figure S5 of the Supporting Information, *ρ* is the measured mass density shown in Table S1, and *C*_p_ is the specific heat. This behavior can be attributed to the evolution of the dominant heat‐transport mechanisms over different temperature ranges. In the low‐ and intermediate‐temperature region (<500 K), the reduction in *κ* is mainly governed by enhanced phonon scattering and carrier scattering, which suppress heat transport. However, at higher temperatures (>500 K), *κ* exhibits a noticeable increase, primarily due to the enhanced bipolar effect. At 303 K, the pristine sample (*x* = 0) exhibits a *κ* of 1.12 W m^−1^ K^−1^. Compared with the matrix sample, the *x* = 0.5 composite shows an increased *κ* of 1.41 W m^−1^ K^−1^. On the one hand, this increase can be partially attributed to the intrinsically high *κ* of ZrB_2_ [64]. On the other hand, it is mainly associated with the increased contribution of *κ*_e_ resulting from the enhanced *σ*. Since the *κ* consists of both electronic and lattice contributions, namely *κ* = *κ*_e_ + *κ*_L_, the enhancement of electrical transport properties inevitably leads to an increase in *κ*_e_. With further increasing ZrB_2_ content, the *κ* begins to decrease. This behavior suggests that higher amounts of ZrB_2_ more effectively suppress the *κ*_L_, thereby offsetting the increase in *κ*_e_.

**FIGURE 6:**
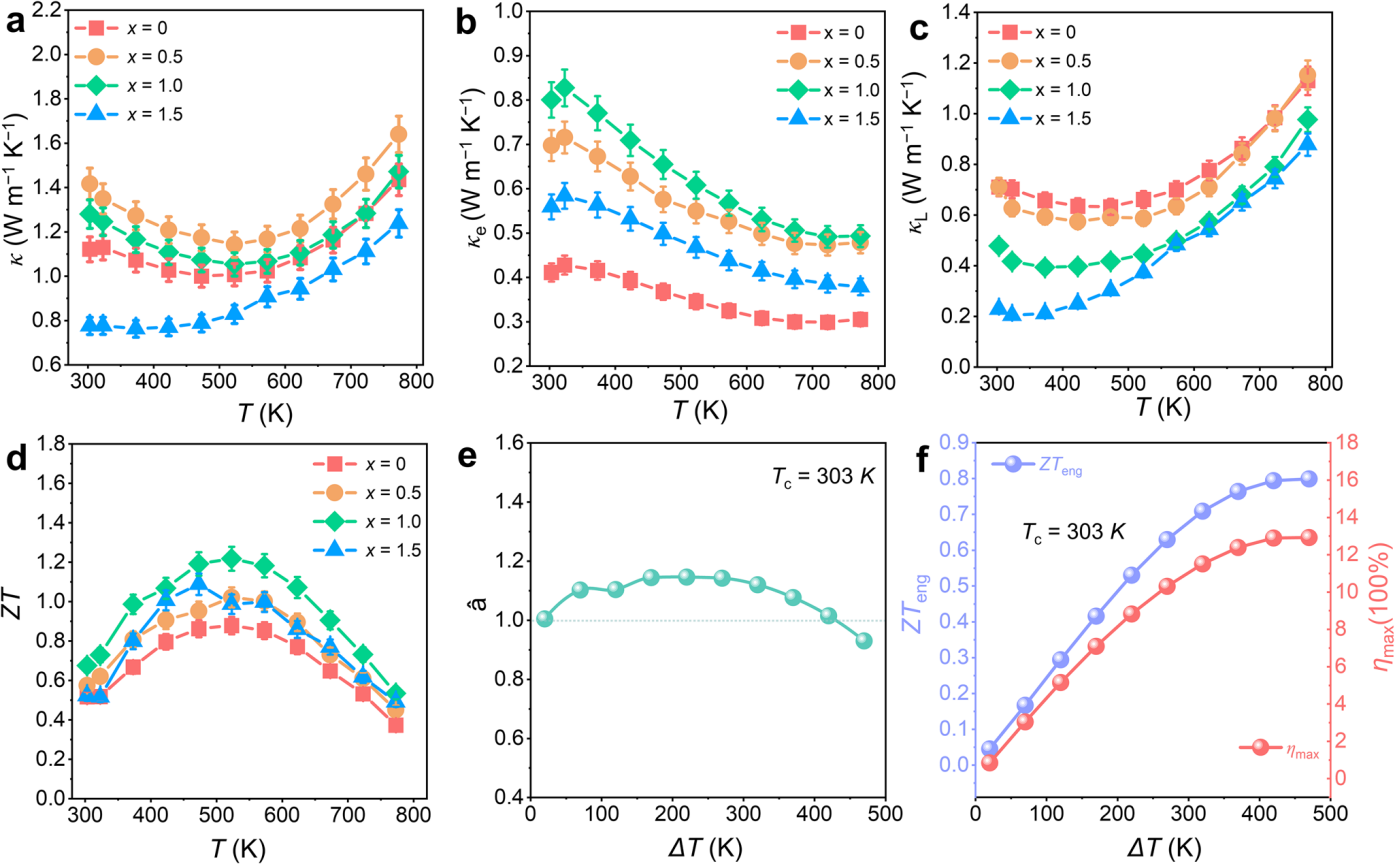
Thermoelectric performance of Mg_3.4_Bi_1.29_Sb_0.7_Te_0.01_‐*x* wt.%ZrB_2_ (*x* = 0, 0.5, 1.0, and 1.5) and calculated device‐relevant parameters of the *x* = 1.0 sample. Temperature‐dependent (a) thermal conductivity (*κ*), (b) electrical thermal conductivity (*κ*_e_), (c) lattice thermal conductivity (*κ*_L_), and (d) *ZT*. (e) Evolution of the dimensionless compatibility factor (*â*) associated with the Thomson effect. (f) Relationship between the calculated engineering *ZT* (*ZT*_eng_) and the theoretical maximum energy‐conversion efficiency (*η*_max_) as a function of temperature difference (Δ*T*).

As shown in Figure 6b, the incorporation of ZrB_2_ significantly increases the *σ* of the composite samples, leading to a pronounced enhancement in the *κ*_e_ compared with the pristine sample. However, the *κ*_L_ exhibits an entirely opposite trend. As illustrated in Figure 6c, the *κ*_L_ is significantly suppressed with increasing ZrB_2_ content. In particular, the *x* = 1.5 sample exhibits the lowest *κ*_L_ value of 0.22 W m^−1^ K^−1^. From the perspective of lattice thermal transport, all ZrB_2_‐containing composite samples exhibit lower *κ*_L_ values than the pristine matrix, and *κ*_L_ decreases progressively with increasing ZrB_2_ content. These results indicate that the introduction of ZrB_2_ effectively suppresses phonon transport. The substantial reduction in *κ*_L_ can be mainly attributed to the ZrB_2_ secondary phase and the large number of heterointerfaces constructed within the composite system. These nanoscale or submicron‐scale interfaces serve as highly effective phonon‐scattering centers, strongly scattering mid‐ and low‐frequency phonons [60]. As a result, the strong phonon‐scattering effect induced by the heterointerfaces successfully compensates for, and even outweighs, the unfavorable increase in *κ*_e_.

Benefiting from the synergistic optimization of electrical and thermal transport properties, the *ZT* of the composite materials is significantly enhanced. As shown in Figure 6d, all composite samples exhibit a general trend in which *ZT* first increases and then decreases with increasing temperature. The pristine sample (*x* = 0) exhibits a relatively low room‐temperature *ZT* value of 0.52. After the incorporation of an appropriate amount of ZrB_2_, however, the *ZT* values are markedly improved across the entire measured temperature range. In particular, the *x* = 1.0 sample achieves the highest thermoelectric performance because an optimal balance is established between interfacial charge regulation and interfacial phonon scattering. As a result, this sample reaches a room‐temperature *ZT* of 0.68 and achieves a peak *ZT* value of 1.22 at 573 K, which is significantly higher than those of the other compositions.

After considering both the electrical and thermal transport contributions, the *n*‐dependent *ZT* was further evaluated using the SPB model, as shown in Figure S6. Near room temperature, the calculated maximum *ZT* for the *x* = 1.0 sample is approximately 0.95 at an optimal *n* ≈ 1.0 × 10^19^ cm^−3^. In comparison, the measured *n* is 4.62 × 10^19^ cm^−3^, placing the sample on the high‐*n* side of the theoretical *ZT* optimum. The corresponding experimental *ZT* is approximately 0.68, which is about 28.4% lower than the calculated maximum. Therefore, a substantial decrease in *n* would be required to shift the sample toward the theoretical *ZT* optimum near room temperature. At 523 K, the calculated maximum *ZT* is approximately 1.32 at *n* ≈ 2.0 × 10^19^ cm^−3^, whereas the measured *n* and experimental *ZT* are 4.63 × 10^19^ cm^−3^ and 1.22, respectively. Although the measured *n* remains higher than the theoretical optimum, the experimental *ZT* is only approximately 7.6% lower than the calculated maximum, indicating that the sample already lies within the broad high‐*ZT* region at 523 K. Thus, further decreasing the *n* would move the sample closer to the exact theoretical optimum at both temperatures, with a greater degree of adjustment required near room temperature. These results indicate that the *n* of the *x* = 1.0 sample is not fully optimized for maximizing *ZT*, but is more favorable for achieving high *ZT* at elevated temperatures than near room temperature. Moreover, the high experimental *ZT* at 523 K should be attributed to the synergistic contributions of favorable electronic transport and suppressed *κ*_L_, rather than to *n* optimization alone. It should also be noted that the optimal *n* predicted by the SPB model are theoretical estimates based on simplified transport assumptions and should not be regarded as exact experimental targets.

To accurately evaluate the power‐generation performance of the device under practical operating conditions, the engineering *ZT* (*ZT*_eng_) and the dimensionless compatibility factor (*â*) were introduced in the calculation of the theoretical maximum conversion efficiency (*η*_max_), thereby accounting for the Thomson effect that is often neglected in conventional constant‐parameter models [58]. Figure 6e,f presents the evolution of the *â*, *ZT*_eng_, and *η*_max_ of the single‐leg device as a function of the operating temperature difference (Δ*T*) under a cold‐side temperature (*T*_c_) of 303 K. As shown in Figure 6f, *ZT*_eng_ exhibits a pronounced monotonic increase with increasing Δ*T* and reaches 0.80 at Δ*T* = 470 K. Meanwhile, Figure 6e shows that the *â* first increases and then gradually decreases with increasing Δ*T*. Notably, within the wide temperature‐difference range of Δ*T* < 420 K, the *â* value remains consistently above 1.0. This result indicates that, within this temperature range, the Thomson effect provides a positive correction to the overall device efficiency, implying that the conventional constant‐parameter model would underestimate the actual conversion efficiency of the material system [65]. When the Δ*T* reaches the maximum value of 470 K, the single‐leg device based on the composite material achieves a theoretical *η*_max_ of approximately 12.93%. This calculated value should be regarded as an upper‐bound estimate of the intrinsic thermoelectric performance rather than the experimentally validated efficiency of a practical device, because device‐level losses, including electrical contact resistance, interfacial thermal resistance, radiative and convective heat dissipation, and geometric effects, were not considered. Nevertheless, the enhanced thermoelectric properties indicate that introducing secondary phases to construct heterointerfaces can effectively regulate charge transport and strengthen phonon scattering. Meanwhile, the compression results in Figure S7 demonstrate that the optimized incorporation of ZrB_2_ improves the mechanical robustness of the matrix, which is beneficial for material processing, device assembly, and structural stability during operation. Therefore, this strategy provides a promising route for simultaneously improving the thermoelectric performance and mechanical reliability of Mg_3_(Sb, Bi)_2_‐based materials, thereby facilitating their potential application in low‐ to medium‐temperature waste‐heat recovery and energy conversion.

## 3 Conclusions

In this work, a Mg_3_(Sb, Bi)_2_/ZrB_2_ heterointerface was constructed by introducing ZrB_2_ as a secondary phase into the Mg_3.4_Bi_1.29_Sb_0.7_Te_0.01_ matrix, thereby achieving synergistic regulation of carrier transport and phonon scattering. First‐principles calculations reveal that the work‐function difference between Mg_3_(Sb, Bi)_2_ and ZrB_2_ can induce interfacial charge redistribution and local potential modulation, thereby promoting an increase in *n*. Experimental results demonstrate that an appropriate amount of ZrB_2_ incorporation can significantly improve the electrical transport performance without noticeably sacrificing *µ*. The optimized 1.0 wt.% ZrB_2_ composite sample exhibits an *S* ^2^ *σ* enhanced from 19.11 µW cm^−1^ K^−2^ for the pristine matrix to 28.51 µW cm^−1^ K^−2^ at room temperature, and further reaches 30.82 µW cm^−1^ K^−2^ at 373 K. Meanwhile, the sample achieves a room‐temperature *ZT* of 0.68 and a peak *ZT* value of 1.22 at 573 K. Based on the *ZT*_eng_, the corresponding *η*_max_ is estimated to reach approximately 12.92% under *T*_c_ = 303 K and a Δ*T* of 470 K. This study demonstrates that conductive ceramic heterointerface engineering based on ZrB_2_ is an effective strategy for enhancing the overall thermoelectric performance of Mg_3_(Sb, Bi)_2_‐based materials in the near‐room‐temperature and mid‐temperature ranges. Moreover, this work provides new insights into interface design for Mg‐based thermoelectric materials.

## 4 Experimental Details

### 4.1 Material Synthesis

All samples were prepared inside a glove box, where both the oxygen and moisture levels were maintained below 0.1 ppm. High‐purity magnesium powder (Mg, 99.95%, Macklin), antimony powder (Sb, 99.99%, Macklin), bismuth powder (Bi, 99.99%, Macklin), tellurium powder (Te, 99.99%, Macklin), and zirconium diboride powder (ZrB_2_, 99.99%, Macklin) were weighed according to the nominal compositions Mg_3.4_Bi_1.29_Sb_0.7_Te_0.01_‐*x* wt.%ZrB_2_ (*x* = 0, 0.5, 1.0, and 1.5). Stainless‐steel milling balls were subsequently added to the powder mixture with a ball‐to‐powder mass ratio of 10:1, followed by sealing under an argon atmosphere. High‐energy ball milling was then carried out at 1200 rpm for 8 h using a high‐speed ball mill (MSK‐SFM‐3, Hefei Kejing Materials Technology Co., Ltd., China). After ball milling, the obtained powders were lightly ground and loaded into a graphite die with a diameter of 12.7 mm lined with graphite foil. The powders were subsequently consolidated by spark plasma sintering (SPS‐3T‐MINI, Shanghai Chenhua Electric Furnace Co., Ltd., China) at 973 K under a pressure of 60 MPa for 5 min to obtain dense bulk samples. The sintered bulks were then cut into appropriate dimensions using a diamond wire saw (STX‐202AQ, Shenyang Kejing Automation Equipment Co., Ltd., China) for subsequent characterizations.

### 4.2 Structural Characterizations

XAFS spectroscopy measurements were performed using a laboratory‐based X‐ray absorption spectrometer (Super XAFS‐H3000, National Innovation Scientific Instruments (Suzhou) Co., Ltd.) in transmission mode at an operating voltage of 35 kV and a current of 25 mA. A Si (8 8 0) spherically bent crystal analyzer with a radius of curvature of 500 mm was employed for the Bi element measurements. The Bi L_3_‐edge X‐ray absorption spectra were collected in transmission mode. All XAFS data were analyzed using the Demeter software package. For all samples, the EXAFS oscillations were extracted from the normalized XAFS spectra by subtracting the atomic background through a quadratic spline fit to the k^2^‐weighted data, where k represents the photoelectron wave vector. The resulting *χ*(k) functions were subsequently Fourier transformed into R‐space. The Fourier‐transform window was selected within the k range of 3–10 Å^−1^. An X‐ray diffractometer (XRD, TD‐3500, Dandong Tongda Technology Co., Ltd., China) was employed to analyze the phase composition and crystal structure of the samples. The measurements were conducted using Cu Kα radiation with a scanning step size of 0.04° and a dwell time of 0.2 s per step. A scanning electron microscope (SEM, FlexSEM1000II, Hitachi High‐Tech Corporation, China) equipped with an EDS was used to characterize the samples and their freshly fractured surfaces in order to evaluate the microstructural features, fracture morphologies, and elemental distributions. For nanoscale structural analysis, the Mg_3.4_Bi_1.29_Sb_0.7_Te_0.01_‐1.0 wt.% ZrB_2_ sample was sectioned using FIB and examined by TEM (FEI Talos F200S, Nari Technology Co., Ltd.).

### 4.3 Performance Testing

The *σ* and *S* of the samples were measured simultaneously over the temperature range of 300–773 K in a low‐pressure helium atmosphere using a four‐probe testing system (CTA‐3, Beijing Creo Technology Co., Ltd., China). The *κ* was calculated according to the relation *κ* = *DρC*_p_, where the *D* was measured using the laser flash method (LFA 467 HT, Netzsch, Germany), the *C*_p_ was estimated based on the Dulong‐Petit law, and the *ρ* was determined using the Archimedes method. The Hall coefficient (*R*_H_) was measured using the van der Pauw method (HMS‐15E, Sichuan Qixu Cryogenic Technology Co., Ltd., China) under a magnetic field of ±0.8 T. The *n* and *µ* were calculated using the relations *n* = 1/(*eR*_H_) and *µ* = *σR*_H_, respectively, where *e* represents the elementary charge. Other key thermoelectric performance parameters, including the *ZT* and the *ZT*_ave_, were calculated based on the above‐mentioned transport parameters. The compressive mechanical properties of the samples were evaluated at room temperature using a universal testing machine (68FM‐300, Instron Corporation, USA). The compressive stress‐strain curves were recorded during loading, and the compressive strength was determined as the maximum compressive stress reached before specimen failure.

### 4.4 DFT Calculations

Density‐functional theory (DFT) calculations are conducted using the projected augmented wave (PAW) method based on the plane wave basis set in the Vienna Ab initio Simulation Package (VASP) [66, 67, 68, 69, 70, 71]. Semi‐local generalized gradient approximation (GGA) with the fully relativistic Perdew‐Burke‐Ernzerhof (PBE) exchange correlation functional is employed to describe the ion–electron interaction of constituent elements [72]. For ionic relaxation, a kinetic energy cutoff of 500 eV is chosen with the Monkhorst‐Pack k‐point mesh spanning less than 0.03/Å^3^ in the Brillouin zone. All ions were allowed to relax in their geometric optimizations until the Hellmann–Feynman force is less than 0.01 eV·Å^−1^. A denser k‐point mesh spanning less than 0.02/Å^3^ is used for calculating non‐self‐consistency, with the convergence criterion of 1×10^−7^ eV. The band structure is calculated along the line‐mode k‐point path based on the Brillouin zone features [73].

### 4.5 SPB Modelling

There are [13, 61, 62]:

(1) $$S\;\left(\eta \right)=\frac{k_{B}}{e}\;\cdot \left[\frac{\left(r+\frac{5}{2}\right)\cdot F_{r+\frac{3}{2}}\left(\eta \right)}{\left(r+\frac{3}{2}\right)\cdot F_{r+\frac{1}{2}}\left(\eta \right)}-\eta \right]$$

(2) $$n=\frac{1}{e\cdot R_{H}}=\frac{{\left(2m^{*}\cdot k_{B}T\right)}^{\frac{3}{2}}}{3\pi^{2}\hbar^{3}}\;\cdot \frac{{\left(r+\frac{3}{2}\right)}^{2}\cdot F_{r+\frac{1}{2}}^{2}\left(\eta \right)}{\left(2r+\frac{3}{2}\right)\cdot F_{2r+\frac{1}{2}}\left(\eta \right)}$$

(3) $$\mu =\left[\frac{e\pi \hbar^{4}}{\sqrt{2}{\left(k_{B}T\right)}^{\frac{3}{2}}}\frac{C_{l}}{{E_{def}}^{2}{\left(m^{*}\right)}^{\frac{5}{2}}}\right]\;\frac{\left(2r+\frac{3}{2}\right)\cdot F_{2r+\frac{1}{2}}\left(\eta \right)}{{\left(r+\frac{3}{2}\right)}^{2}\cdot F_{r+\frac{1}{2}}\left(\eta \right)}$$

(4) $$\begin{array}{c} L={\left(\frac{k_{B}}{e}\right)}^{2}\;\cdot \left\{\frac{\left(r+\frac{7}{2}\right)\cdot F_{r+\frac{5}{2}}\left(\eta \right)}{\left(r+\frac{3}{2}\right)\cdot F_{r+\frac{1}{2}}\left(\eta \right)}-{\left[\frac{\left(r+\frac{5}{2}\right)\cdot F_{r+\frac{3}{2}}\left(\eta \right)}{\left(r+\frac{3}{2}\right)\cdot F_{r+\frac{1}{2}}\left(\eta \right)}\right]}^{2}\right\} \end{array}$$

where *η* is the reduced Fermi level, *k*_B_ is the Boltzmann constant, *r* is the carrier scattering factor (*r* = −1/2 for acoustic phonon scattering), *R*_H_ is the Hall coefficient, *C*_l_ is the elastic constant for longitudinal vibrations, and *E*_def_ is the deformation potential coefficient, respectively. There is [74, 75, 76, 77]:

(5) Cl=vl2·ρ

The longitudinal velocity *v*_l_ is derived from the reference [78, 79, 80]. *F_i_* (*η*) is the Fermi integral, expressed as [74, 75, 76, 77]:

(6) $$F_{i}\;\left(\eta \right)=\int_{0}^{\infty }\frac{x^{i}}{1+e^{\left(x-\eta \right)}}\;dx$$

### 4.6 Energy Conversion Efficiency Calculation

The traditional *ZT*_ave_ is calculated as follows [81]:

(7) $$ZT_{ave}=\frac{\int_{T_{c}}^{T_{h}}ZT\left(T\right)dT}{T_{h}-T_{c}}\;$$

Since *S*, *σ*, and *κ* are usually assumed to be temperature‐independent in traditional calculations, the accuracy of using *ZT*_ave_ to determine the conventional *η*_max_ is low. In order to solve this problem, researchers have developed a *ZT*_eng_, which is calculated as follows [82, 83]:

(8) $$ZT_{eng}=\frac{{\left[\int_{T_{c}}^{T_{h}}S\left(T\right)dT\right]}^{2}\left[\int_{T_{c}}^{T_{h}}\sigma \left(T\right)dT\right]}{\left[\int_{T_{c}}^{T_{h}}\kappa \left(T\right)dT\right]}\;\Delta T$$

The formula for calculating the *η*_max_ based on *ZT*_eng_ is as follows [82, 83]:

(9) $$\eta_{max}=\varepsilon_{c}\;\frac{\sqrt{1+ZT_{eng}\left(\frac{\hat{a}}{\varepsilon_{c}}-\frac{1}{2}\right)}-1}{\hat{a}\sqrt{1+ZT_{eng}\left(\frac{\hat{a}}{\varepsilon_{c}}-\frac{1}{2}\right)}-\varepsilon_{c}}$$

The *â* is calculated as follows [58]:

(10) $$\hat{a}=S\left(T_{h}\right)\Delta T/\left[\int_{T_{c}}^{T_{h}}S\left(T\right)dT\right]$$

In the formula, *T*_h_ represents the hot end temperature.

### 4.7 Uncertainty Analysis

In evaluating the performance of thermoelectric materials, the measurement accuracy and data reliability of the three key transport parameters, *σ*, *S*, and *κ*, are of critical importance. To comprehensively and reliably assess the measurement uncertainty and data reproducibility, a rigorous testing protocol was adopted. Each sample was measured at least three times independently to ensure the consistency and reproducibility of the obtained results. In this study, repeated measurements revealed that the *σ* exhibited fluctuations of approximately ±10% around the average value. Such variations mainly originate from minor differences in sample preparation, instrumental limitations, and slight environmental fluctuations during testing. In contrast, the *S* demonstrated relatively higher stability, with measurement variations within ±5%, indicating excellent accuracy and reproducibility. Similarly, the *κ* exhibited a fluctuation range of approximately ±5%, further confirming the reliability and consistency of the thermal transport measurements. Other key thermoelectric performance parameters, such as the *ZT*, were subsequently derived from these three fundamental transport parameters using standard equations.

## Author Contributions

**Meng Li**: software. **Yangyang Xu**: methodology, software, visualization, writing – original draft. **Li Zhang**: conceptualization, formal analysis, supervision, resources, project administration, validation, investigation, funding acquisition. **Yan‐Ling Yang**: project administration. **Jie Zhang**: methodology, formal analysis. **Nan‐Hai Li**: formal analysis, software. **Zhi‐Gang Chen**: conceptualization, investigation, validation, writing – review and editing, formal analysis, project administration, data curation, supervision. **Xiao‐Lei Shi**: conceptualization, data curation, formal analysis, project administration, supervision, validation, visualization, investigation, writing – review and editing.

## Conflicts of Interest

The authors declare no competing interests.

## Supporting information




**Supporting File**: advs77148‐sup‐0001‐SuppMat.docx.

## Data Availability

All data associated with the study are included in the article and the Supporting Information. Additional information is available from the Lead Contact upon reasonable request.
