# Supplementary material for: Enhancing Near‐Room‐Temperature Thermoelectric Performance of n‐Type Mg3(Sb, Bi)2‐Based Materials via ZrB2 Heterointerface Engineering
Source: Adv Sci (Weinh). 2026 Aug 13:e77148. Online ahead of print. doi: 10.1002/advs.77148 (PMC13472121; doi:10.1002/advs.77148)
Supplement: Supplementary file 1 [file ADVS-9999-e77148-s001.docx]

**Supporting Information**

Enhancing Near-Room-Temperature Thermoelectric Performance of n-Type Mg_3_(Sb, Bi)_2_-Based Materials *via* ZrB_2_ Heterointerface Engineering

*Yangyang Xu,^1^ Li Zhang,^1,*^ Meng Li,^2^ Jie Zhang,^1^ Nan-Hai Li,^2^ Yan-Ling Yang,^1^ Xiao-Lei Shi,^2,*^ Zhi-Gang Chen^2,*^*

^1^ School of Materials Science and Engineering, Shaanxi Key Laboratory of Green Preparation and Functionalization for Inorganic Materials, Shaanxi University of Science & Technology, Xi’an 710021, P.R. China

^2^ School of Chemistry and Physics, ARC Research Hub in Zero-emission Power Generation for Carbon Neutrality, and Centre for Materials Science, Queensland University of Technology, Brisbane, Queensland 4000, Australia

Email: zhangli@sust.edu.cn (L. Zhang), xiaolei.shi@qut.edu.au (X.-L. Shi), zhigang.chen@qut.edu.au (Z.-G. Chen)

**Keywords**: thermoelectric, Mg_3_(Sb, Bi)_2_, ZrB_2_, interface, material.

**Supporting Figures**

**Figure S1.** Differential charge density distribution of the adsorption system. The yellow and cyan isosurfaces represent electron accumulation and depletion, respectively, indicating pronounced charge redistribution and interfacial interaction between the adsorbates and the substrate. Green, orange, and purple spheres denote different atomic species. The isosurface value is set to ±0.00170971 e Å^−3^.

**Figure S2**. Low-magnification transmission electron microscopy (TEM) and elemental distribution of the Mg_3.4_Bi_1.29_Sb_0.7_Te_0.01_-1.0 wt.% ZrB_2_ sample. **(a)** Low-magnification TEM image of the focused ion beam (FIB)-prepared lamella, showing the overall microstructure and grain-boundary distribution. **(b)** Corresponding TEM energy dispersive X-ray spectroscopy (EDS) elemental maps of Zr, B, Sb, Mg, Bi, and Te obtained from the region shown in (a).

**Figure S3.** TEM characterization of an MgO secondary phase in the Mg_3.4_Bi_1.29_Sb_0.7_Te_0.01_-1.0 wt.% ZrB_2_ sample. **(a)** TEM image of a representative MgO particle embedded in the Mg_3_(Sb, Bi)_2_-based matrix. **(b)** Corresponding TEM-EDS elemental maps of Mg, O, Te, Bi, and Sb, showing the co-enrichment of Mg and O within the particle and the depletion of matrix elements.

**Figure S4.** **Single parabolic band (SPB)-model-calculated carrier-concentration (*n*) dependence of the thermoelectric properties of Mg_3.4_Bi_1.29_Sb_0.7_Te_0.01_-*x* wt.% ZrB_2_ (*x* = 0 0.5, 1.0, and 1.5).** Power factor (*S*^2^*σ*) as a function of *n* at **(a)** 303 K and **(b)** 523 K. The curves and symbols denote the SPB-calculated and experimental results, respectively.

**Figure S5.** Temperature-dependent thermal diffusivity (*D*) of Mg_3.4_Bi_1.29_Sb_0.7_Te_0.01_-*x* wt%ZrB_2_ (*x* = 0, 0.5, 1.0 and 1.5).

**Figure S6.** **SPB-model-calculated *n* dependence of the thermoelectric properties of Mg_3.4_Bi_1.29_Sb_0.7_Te_0.01_-*x* wt.% ZrB_2_ (*x* = 0 0.5, 1.0, and 1.5).** *ZT* as a function of *n* at (a) 303 K and (b) 523 K. The curves and symbols denote the SPB-calculated and experimental results, respectively.

**Figure S7.** Compressive stress-strain curves of Mg_3.4_Bi_1.29_Sb_0.7_Te_0.01_-*x* wt% ZrB_2_ (*x* = 0, 0.5, 1.0 and 1.5)

**Supporting Table**

**Table S1.** Bulk, theoretical, and relative densities of Mg_3+_*_x_*Bi_1.29_Sb_0.7_Te_0.01_-*x* wt.% ZrB_2_ composite samples.

| **ZrB_2 ­­_content, *x* (wt%)** | **Bulk density,**  ***ρ*_bulk_ (g cm^−3^)** | **Theoretical density,**  ***ρ*_th_ (g cm^−3^)** | **Relative density,**  ***ρ*_rel_ (%)** |
| --- | --- | --- | --- |
| 0 | 4.929 | 5.438 | 90.64 |
| 0.5 | 4.997 | 5.441 | 91.84 |
| 1.0 | 5.021 | 5.444 | 92.23 |
| 1.5 | 4.968 | 5.447 | 91.21 |
